# ARIA guideline 2019: treatment of allergic rhinitis in the German health system 

**DOI:** 10.5414/ALX02120E

**Published:** 2019-12-30

**Authors:** Ludger Klimek, Claus Bachert, Oliver Pfaar, Sven Becker, Thomas Bieber, Randolph Brehler, Roland Buhl, Ingrid Casper, Adam Chaker, Wolfgang Czech, Jörg Fischer, Thomas Fuchs, Michael Gerstlauer, Karl Hörmann, Thilo Jakob, Kirsten Jung, Matthias V. Kopp, Vera Mahler, Hans Merk, Norbert Mülleneisen, Katja Nemat, Uta Rabe, Johannes Ring, Joachim Saloga, Wolfgang Schlenter, Carsten Schmidt-Weber, Holger Seyfarth, Annette Sperl, Thomas Spindler, Petra Staubach, Sebastian Strieth, Regina Treudler, Christian Vogelberg, Andrea Wallrafen, Wolfgang Wehrmann, Holger Wrede, Torsten Zuberbier, Anna Bedbrook, Giorgio W. Canonica, Victoria Cardona, Thomas B. Casale, Wienczylawa Czarlewski, Wytske J. Fokkens, Eckard Hamelmann, Marek Jutel, Désirée Larenas-Linnemann, Joaquim Mullol, Nikolaos G. Papadopoulos, Sanna Toppila-Salmi, Thomas Werfel, Jean Bousquet

**Affiliations:** 1 Center of Rhinology and Allergology, Wiesbaden, Germany,; 2 Upper Airways Research Laboratory and Department of Oto-Rhino-Laryngology, Ghent University and Ghent, University Hospital, Ghent, Belgium, Division of ENT Diseases, CLINTEC, Karolinska Institute, University of Stockholm, Stockholm, Sweden,; 3 Department of Otorhinolaryngology, Head and Neck, Surgery, Section of Rhinology and Allergy, University, Hospital Marburg, Philipps-Universität Marburg,Marburg, Germany,; 4 Department of Otolaryngology, Head and Neck Surgery, University of Tübingen, Tübingen, Germany,; 5 Department of Dermatology and Allergy, University of Bonn, Bonn, Germany, Christine Kühne-Center for Allergy Research and Education (CK-CARE) Davos-Augsburg-Bonn-St Gallen-Zürich, St. Gallen, Switzerland,; 6 Department of Allergy, Occupational Dermatology and Environmental Medicine, Universitätsklinikum Münster, Münster, Germany,; 7 Pulmonary Department,Mainz University Hospital,Mainz, Germany,; 8 Department of Otolaryngology and Center for Allergy and Environment (ZAUM), Klinikum rechts der Isar, Technical University of Munich and Helmholtz Center Munich, Munich, Germany,; 9 Department of Dermatology, University of Freiburg, Freiburg, Germany,; 10 Department of Dermatology, Eberhard Karls University, Tübingen, Tübingen, Germany,; 11 Department of Dermatology, Venereology, and Allergology, University Medical Center, Georg August University, Göttingen, Germany,; 12 Pediatric Pneumology and Allergology Unit, Medical University of Augsburg, Augsburg, Germany,; 13 Department of Otorhinolaryngology, Mannheim University Hospital, Mannheim, Germany,; 14 Department of Dermatology and Allergology, University Medical Center Gießen and Marburg, Campus Gießen, Justus-Liebig-University, Gießen, Germany,; 15 Group Practice for Dermatology, Erfurt, Germany,; 16 Clinic of Pediatric and Adolescent Medicine, Airway Research Center North (ARCN), Member of the German Lung Center (DZL), Lübeck University, Lübeck, Germany,; 17 Medical Faculty, Friedrich-Alexander-University (FAU) Erlangen-Nürnberg, Germany,; 18 Department of Dermatology and Allergology, University Hospital, RWTH Aachen University, Aachen, Germany,; 19 Asthma and Allergy Centre, Leverkusen, Germany,; 20 Department of Pediatrics, University Hospital Carl Gustav Carus, Technical University of Dresden, Dresden, Germany,; 21 Department of Allergology, Johanniter-Krankenhaus im Fläming Treuenbrietzen GmbH, Treuenbrietzen, Germany,; 22 Department and Outpatient Clinic for Dermatology and Allergology am Biederstein, Technical University of Munich, Munich, Germany and Christine Kühne Center for Allergy Research and Education (CK-Care), Davos, Switzerland,; 23 Department of Dermatology, University Medical Center Mainz,Mainz, Germany,; 24 Former Head ENT - Department, Katharina-Kasper-Kliniken, Marienkrankenhaus, c/o University Hospital, Frankfurt, Germany,; 25 Center for Allergy and Environment (ZAUM), Member of the German Center of Lung Research (DZL) and the Inflammation and Immunology Helmholtz Initiative, Technical University of Munich and Helmholtz Center Munich, Munich, Germany,; 26 Pharmacy Association in Hesse, Offenbach, Germany,; 27 Allergy Campus Davos, Hochgebirgsklinik Davos dpt. Pediatrics, Davos, Switzerland,; 28 Department of Otolaryngology, University Medical Center Mainz, Mainz, Germany,; 29 Department of Dermatology, Venereology and Allergology, LICA – Leipzig Comprehensive Allergy Center, University of Leipzig, Leipzig, Germany,; 30 Department of Pediatric Pneumology and Allergology, University Hospital Carl Gustav Carus, Technical University of Dresden, Dresden, Germany,; 31 German Allergy and Asthma Association, Mönchengladbach, Germany,; 32 Dermatology Group Practice,Münster, Germany,; 33 Herford, North Rhine-Westphalia, Germany,; 34 Department of Dermatology and Allergy, Allergie-Centrum – Charité, Charité – Universitätsmedizin, Berlin, Berlin, Germany,; 35 MACVIA-France, Montpellier, France,; 36 Allergy Section, Allergy and Respiratory Diseases, DIMI, University of Genoa, Genoa, Italy,; 37 Department of Internal Medicine, Hospital Universitari Vall d’Hebron, Barcelona, Spain,; 38 Division of Allergy and Immunology, University of South Florida, Tampa, FL, USA,; 39 Medical Consulting Czarlewski, Levallois, France,; 40 Department Otorhinolaryngologie, Academic Medical Centers, Amsterdam, The Netherlands,; 41 Children’s Center, Protestant Hospital Bethel, University Bielefeld, Bielefeld Germany,; 42 Department of Clinical Immunology, Wroclaw Medical University, Wroclaw, Poland and ALL-MED Medical Research Institute, Wroclaw, Poland,; 43 HospitalMédica Sur, México City, Mexico,; 44 Unitat de Rinologia i Clínica de l’Olfacte, Servei d’ORL, Hospital Clínic, Clinical and Experimental Respiratory Immunoallergy, IDIBAPS, University of Barcelona, Barcelona, Spain,; 45 Department of Allergy, 2; 46 Pediatric Clinic, University of Athens, Athens, Greece,; 47 Haartman Institute, University of Helsinki, Helsinki, Finland,; 48 Division of Immunodermatology and Allergy Research, Department of Dermatology and Allergy, Hannover Medical School, Hannover, Germany,; 49 University Hospital, Montpellier, 49 INSERM, Unit 1168, Paris, France

**Keywords:** allergic diseases, allergic asthma, integrated care pathway, allergen-specific immunotherapy, health care system

## Abstract

Background: The number of patients affected by allergies is increasing worldwide. The resulting allergic diseases are leading to significant costs for health care and social systems. Integrated care pathways are needed to enable comprehensive care within the national health systems. The ARIA (Allergic Rhinitis and its Impact on Asthma) initiative develops internationally applicable guidelines for allergic respiratory diseases. Methods: ARIA serves to improve the care of patients with allergies and chronic respiratory diseases. In collaboration with other international initiatives, national associations and patient organizations in the field of allergies and respiratory diseases, real-life integrated care pathways have been developed for a digitally assisted, integrative, individualized treatment of allergic rhinitis (AR) with comorbid asthma. In the present work, these integrated care pathways have been adapted to the German situation and health system. Results: The present ICP (integrated care pathway) guideline covers key areas of the care of AR patients with and without asthma. It includes the views of patients and other healthcare providers. Discussion: A comprehensive ICP guideline can reflect real-life care better than traditional guideline models.

**First published in Allergo J Int, Vol. 28, 2019, pp. 255-276 DOI 10.1007/s40629-019-00110-9** [Table Abbreviations]

## Introduction 

Worldwide, both the number of patients affected by allergies and the costs of allergic diseases are increasing rapidly. Strategies are needed to transfer integrated care pathways (ICPs) into national health systems [18]. 

**Figure 1.**
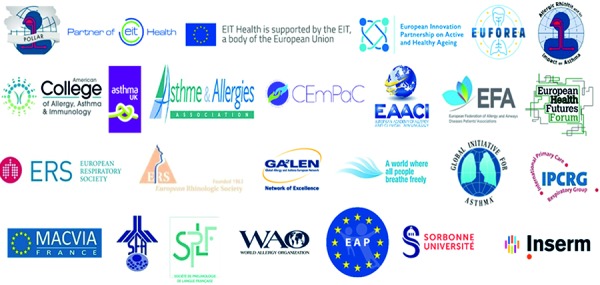
Organizations supporting the ARIA meeting in Paris.

**Figure 2.**
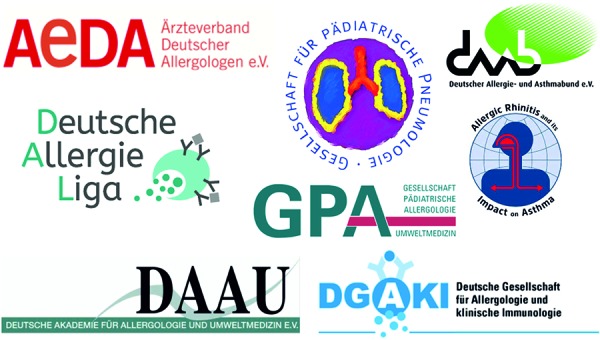
German organizations supporting this publication together with the German ARIA group.

A meeting on chronic disease care has been held in Paris (December 3, 2018). The event was organized by MASK (Mobile Airways Sentinel NetworK) [19] and POLLAR (Impact of Air POLLution on Asthma and Rhinitis, EIT Health) [20], in collaboration with professional and patient organizations in the field of allergy and airway diseases (Figure 1). The evaluation of real-life integrated care pathways (ICPs) was recommended for digitally enabled, integrated, personalized care for rhinitis and asthma multimorbidity and environmental exposure was embedded [18, 19]. This publication represents an adaptation of this real-life ICP to the German health care system and is supported by the organizations and associations listed in Figure 2. 

### Information on the burden and costs of allergic diseases, epidemiology and medication use in Germany 

**Figure 3.**
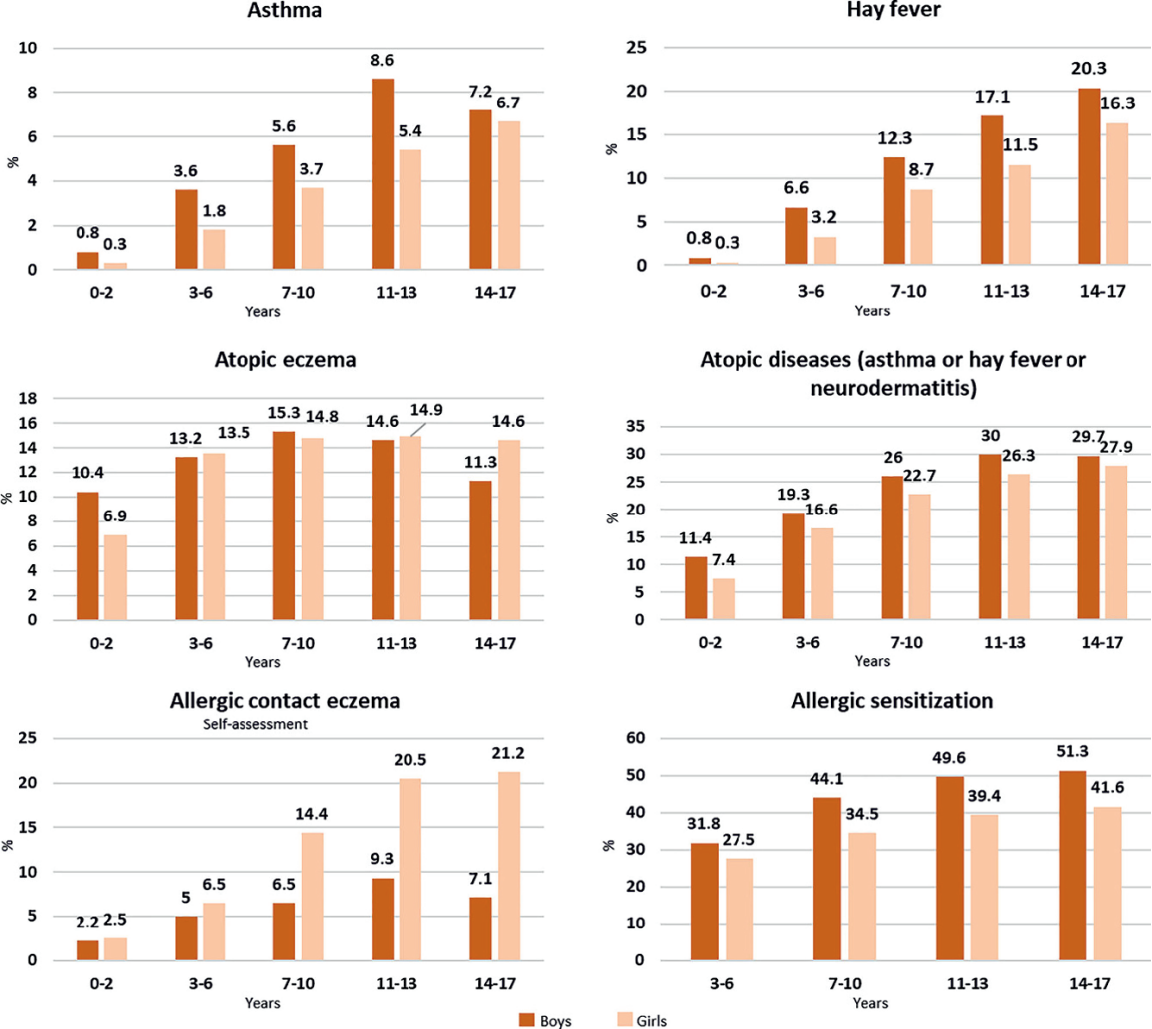
Lifetime prevalence (in %) of common allergic diseases and point prevalence (in %) of allergic sensitizations in children and adolescents in Germany. Results of the KiGGS baseline survey 2003 – 2006. (Reprinted with kind permission from [22]).

The incidence of allergies in Germany has risen rapidly since the 1970s. Approximately 30 million people are affected by allergic diseases (Figure 3; [21]). Recent figures on the 12-month prevalence of allergies have been published by the Robert Koch Institute in the *Journal of Health Monitoring* (Figure 3; [22]). Here, 28.1% of adults were reported as being currently affected by allergies. Women (31.6%) were significantly more affected than men (24.5%). In addition, younger and middle-aged adults (up to 65 years) reported allergies more often than the elderly. In childhood and adolescence, allergic diseases were even the most common health problems. In the course of time, the authors noted that, above all, the proportion of children up to 6 years with asthma and hay fever increased [22]. Early hay fever increased the risk of asthma by 3.6 times in boys and by 2.3 times in girls. The authors of the Robert Koch Institute report concluded that these data support the demand for early causal treatment of hay fever, as the risk of the allergic march is at its greatest when hay fever develops in early childhood [22]. 

**Figure 4.**
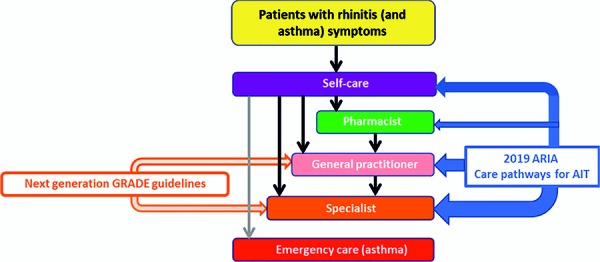
The next-generation ARIA care pathways considered in this publication. (Reprinted with kind permission from [27]).

ICPs are structured, multidisciplinary care plans that describe key steps in patient care [23]. They promote the implementation of guideline recommendations into local protocols and their application in clinical practice [24, 25]. Typically, ICPs improve recommendations by iteratively combining interventions, integrating quality assurance, and promoting the coordination of treatment. AIRWAYS ICPs (Integrated Care Pathways for Airway Diseases) [26] were the first steps in the development of ICPs for patients with rhinitis and asthma as a comorbidity, or for patients with multimorbidities. New guidelines for pharmacotherapy and ICPs for allergen-specific immunotherapy (AIT) are currently being developed for allergic rhinitis (AR). Following the Paris meeting, two separate documents were produced [27, 28]. The present publication is a summary of these documents and transfers them to the German health system (Figure 4). In the future, this adaptation will also be carried out for various other countries and regions in order to adapt the results to the local conditions and corresponding national health systems. 

## Next-generation ARIA-GRADE guidelines 

Pharmacotherapy for AR patients is considered to control the disease. It depends on (i) patient empowerment and preferences, (ii) prominent symptoms, symptom severity and multimorbidity, (iii) efficacy and safety of the treatment [29], (iv) speed of onset of action of treatment, (v) current treatment, (vi) historic response to treatment, (vii) impact on sleep and work productivity [30, 31], (viii) self-management strategies and (ix) resource use. 

**Figure 5.**
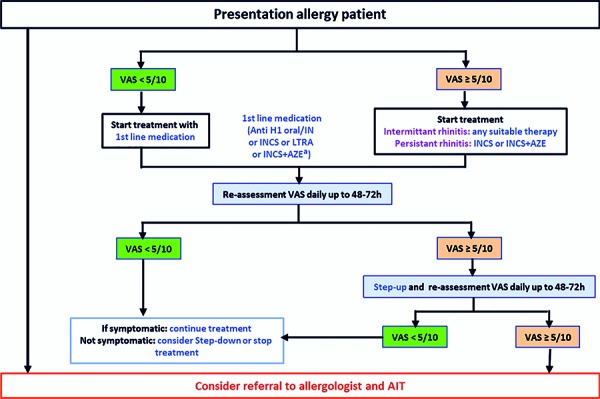
Step-up algorithm in untreated patients (adolescents over 12 years and adults) based on visual analogue scales. The proposed algorithm considers the patient’s preferences: If ocular symptoms persist after initiation of treatment, local conjunctival therapy should be added. Due to the characteristics in the German health care system of direct specialist access, the entire treatment chain from anamnesis, to allergen avoidance, pharmacological therapy, indication and implementation of AIT can also be performed by an allergologically competent specialist or a physician with additional training in allergology, which enables an early AIT. (Reprinted with kind permission from [32]).

**Figure 6.**
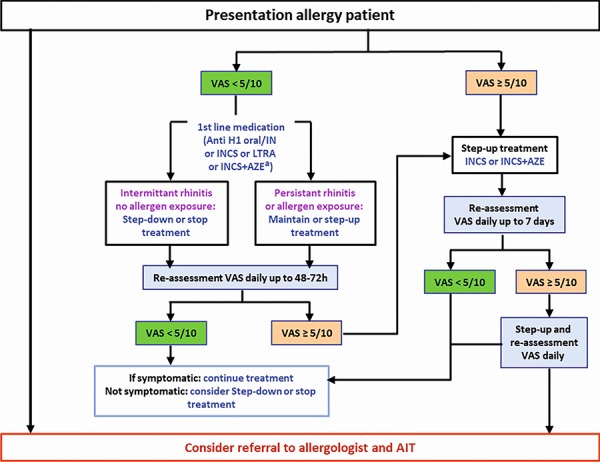
Step-up algorithm in treated patients (adolescents over 12 years and adults) based on visual analogue scales. The proposed algorithm considers the patient’s preferences: If ocular symptoms persist after initiation of treatment, local conjunctival therapy should be added. Due to the characteristics in the German health care system of direct specialist access, the entire treatment chain from anamnesis, to allergen avoidance, pharmacological therapy, indication and implementation of AIT can also be performed by an allergologically competent specialist or a physician with additional training in allergology, which enables an early AIT. (Reprinted with kind permission from [32]).

An algorithm was devised [32] and digitalized [33] to propose step-up or step-down AR treatment (Figure 5, 6). The guideline group aims to adapt this algorithm to the availability of medicines and resources in different countries. Moreover, algorithms require testing via randomized controlled trials (RCTs) and observational research called real-world evidence (RWE) [34, 35, 36]. 

National and international guidelines are mostly based on the database of randomized controlled trials (RCTs). In fact, the GRADE method (Grading of Recommendations, Assessment, Development and Evaluation) explicitly takes into account all types of study designs, from RCTs to observational studies and case reports [37, 38, 39]. GRADE also considers data on preferences, acceptability and feasibility or accuracy of results. 

For the applicability of guidelines in the routine care of patients, the results of RCTs are, in part, limited by the parameters of clinical trials [40]. Therefore, information from real-world evidence (RWE) is increasingly being considered in the creation of practice- oriented guidelines. Ideally, both approaches will be merged [4]. 

During the Paris meeting, *next-generation recommendations* were developed leading to a GRADE-based guideline for the pharmacological treatment of AR [3, 4, 5, 32]. These recommendations were tested with RWE using the MASK-air health app [19, 41]. The algorithm proposed by the consensus group is based on a summary of all this information [32]. In this publication, these recommendations are adapted to the situation of the German health care system. 

**Care-relevant evaluation of drugs for the treatment of allergic rhinitis**

Over the counter (OTC) medicines cannot generally be prescribed at the expense of the statutory health insurance (SHI) of the German health care system. The majority of AR drugs, such as many antihistamines, numerous INCSs (intranasal corticosteroids), or alpha sympathomimetics or low-effective mast cell stabilizers, are nonprescription drugs. They cannot therefore be prescribed at the expense of the statutory health insurance to adolescents from 12 years on and to adults according to Annex I of the pharmaceutical directives (Arzneimittel-Richtlinien (AMR)) (Infobox 1). 

According to the specifications of many SHI pharmacotherapy consultants, OTC preparations should preferably be prescribed on a green prescription or should only be recommended. As a rule, the costs for nonprescription medicines are borne by the insured persons themselves. However, exceptions apply to seriously ill AR patients and should be considered so that these patients with severe disease can be treated under medical supervision. 

Exceptions apply to OTC preparations which are used as the standard therapy for serious diseases for children up to the age of 12 and adolescents with developmental disabilities up to the age of 18 years. 

According to the OTC exemption list in Annex I of the Pharmaceutical Directive, the serious diseases in which nonprescription antihistamines can be prescribed for special cases are:

- only in emergency kits for treatment of bee, wasp, hornet venom allergies
- only for the treatment of severe, recurrent urticaria
- only in severe, persistent pruritus
- only for the treatment of severe allergic rhinitis,
- where topical nasal treatment with glucocorticosteroids is not sufficient.

In these cases, nonprescription antihistamines can also be the economic alternative, regardless of age. 

Intranasal glucocorticosteroids (INCSs) are the gold standard in the pharmacological therapy of AR, as also outlined in the results of the Paris ARIA conference. 

Since October 15, 2016, however, many INCSs can no longer be prescribed on a red SHI prescription for adult patients with Seasonal AR. Specifically, this affects beclometasone, fluticasone and mometasone with their esters under the following conditions:

- The medication may only be given by a doctor after the first diagnosis of seasonal allergic rhinitis
- A maximum daily dose of 400/200mg must be maintained
- Containers and outer shells must provide appropriate information
- The medicines may only be given to adults

Exemptions exist for serious disorders affecting quality of life. In August 2018, the Joint Federal Committee (J-FC) decided that it is once again possible to prescribe INCSs with the active ingredient beclomethasone, fluticasone and mometasone at the expense of the statutory health insurance *“for the treatment of persistent allergic rhinitis with severe symptoms”.*

In addition, the J-FC acknowledged that serious forms of AR – permanently impairing quality of life in the long term due to severity of the disorder – are a serious disease within the meaning of the Pharmaceutical Directive. 

**Figure 7.**
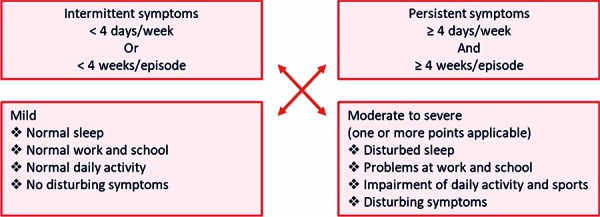
Assessment of the ability of prescription of antihistamines and INCSs in AR. This is possible in cases of persistent, serious AR at the expense of the SHI.

AR is considered serious “if it is a persistent allergic rhinitis” in which the symptoms occur “at least 4 days a week and over a period of at least 4 weeks” and must therefore be classified as severe. The J-FC followed this definition from an earlier ARIA guideline for its supporting reasons (Figure 7; [18, 42]). 

If there are no serious symptoms or if the symptoms are present for less than 4 weeks, patients must pay for the product themselves. 

Furthermore, the conditions for the prescription of nonprescription antihistamines for patients with SHI have been adjusted in the wording. Again, it must be a *“persistent allergic rhinitis with serious symptoms”.*

To date, in Germany, there is no arrangement for SHI patients with severe AR symptoms, for whom antihistamines and INCSs are not effective. These patients usually use arbitrary combinations of different preparations and drug groups, whereas only the fixed combination MPAzeFlu (combined intranasal FP and azelastine (Aze) in a nasal spray) has evidence-based efficacy in the therapeutic area. Currently, in Germany, no generic drugs exist for fixed combinations, and there is no possibility of OTC use, since the fixed combinations were not exempted from the prescription. A distinction of these versus free and arbitrary combinations of active ingredients through the J-FC and the SHI would be desirable, because the latter drug combinations do not hold proof of efficacy from controlled clinical trials. Moreover, contrary evidence exists that the simultaneous use of an oral H1-antihistamine and INCSs has no better effectiveness than INCSs alone [3, 4]. 

### Basic principles for the development of ARIA ICPs 

**MASK algorithm for the pharmacological treatment of AR**

The MASK algorithm, based on the visual analogue scale (VAS) [43], was developed by the ARIA Expert Group for the selection of pharmacotherapy and the gradual step-up or step-down of therapy depending on symptom control ([32]; Figure 5, Figure 6). 

**Revision of ARIA 2010, 2016 and US Practice Parameters 2017**

Although only few direct comparative drug studies are available in RCTs [11, 12, 44, 45], a comparison of AR drugs has been made in several reviews [29] and guidelines [3, 4, 5, 32]. In one review, a similar potency was assumed for AR drugs [46]. But this study used a methodology that did not allow for distinction between drugs. However, the AR GRADE Guidelines agree in some important respects [3, 4, 5, 32] (Infobox 2): 

The revision of the ARIA Guideline 2016 [3] and the US Practice Parameters 2017 [4], which were developed independently, used the same methodological approach with GRADE [37, 38, 39]. Interestingly, identical questions were analyzed. In the treatment of moderate to severe rhinitis, two main factors were considered: effectiveness and onset of action (Infobox 3 and Infobox 4). However, for all these recommendations, the evidence level is low (2 and 3) or very low (1). The ARIA 2016 revision [3] and the US Practice Parameters 2017 [4], which are mainly based on RCTs, support the MASK algorithm [32]. 

**Onset of action of the medicines**

There are three types of studies to evaluate the onset of action of AR drugs [47, 48]: (i) the standard doubleblind phase III RCT, (ii) park setting studies and (iii) allergen exposure chamber (AEC) studies [49]. The RCTs usually provide information about the efficacy of the investigational product versus placebo but are not designed to capture the exact minute of the onset of action. On the other hand, AECs offer several advantages for evaluating the onset of medication, which can be detected to the minute [49]. Furthermore, data from AEC studies are considered to be more robust than those from park studies [50]. 

Several nasal drugs were tested in the pollen exposure chambers of Ontario [16, 51, 52, 53] and Vienna [54, 55, 56]. Ontario’s chamber studies show the rapid onset of action of azelastine and its combinations, including MPAzeFlu. Other intranasal H1 antihistamines showed a slower onset of action. However, intranasal corticosteroids (INCSs) (alone or with oral H1 antihistamines) did not show an onset of action for 2h. The Vienna Chamber studies show that azelastine and levocabastinin combined with fluticasone furoate are the fastest acting drugs in comparison to oral H1-antihistamines or ICNSs alone [54, 55, 56]. 

**Real-life studies using mHealth/health apps**

The next-generation ARIA guidelines tested the GRADE recommendations with RWE based on data from mHealth-tools to confirm or refine the guidelines and the MASK algorithm. Although many mHealth tools are available for AR [57], MASK has unique data on pharmacotherapy that can be used in RWE [19, 58]. 

**2017 MASK treatment study** A pilot study using a cross-sectional real-world observational design with 2,871 users (17,091 days of VAS) provided insights into real-life AR treatment using VAS for overall allergic symptoms (VAS-global) in 15 countries [41] (Infobox 5). 

**2017 MASK treatment study [** 59 **]** A cross-sectional real-world observational study was conducted in 22 countries to complement the 2016 pilot study [41]. A total of 9,122 users filled in 112,054 days of VAS in 2016 and 2017. The same results were observed for VAS-global. Moreover, the same trend was found for VAS nasal symptoms, asthma, eye symptoms and work productivity (Infobox 5). 

**2018 MASK treatment adherence study [** 60 **]** An observational cross-sectional study was carried out on 12,143 users. Adherence is impossible to prove directly as users do not report data every day and may not report all medications used. Secondary adherence was assessed using modified Medication Possession Ratio (MPR) and Proportion of Days Covered (PDC). Adherence was lower than 5%. 

**Limitations of MASK** As for all studies using participatory data, potential biases include the likelihood of sampling bias and outcome misclassification that cannot be assessed and, due to ethical problems, availability of very little information on patient (or day) characteristics. App users are not representative of all patients with rhinitis. 

MASK used days in a cross-sectional analysis [41, 61] because there was no clear pattern of treatment. Furthermore, a longitudinal study was not feasible since patients mostly use the App intermittently. The diagnosis of AR was not supported by a physician but it is likely that most users were suffering from rhinitis (allergic or nonallergic) [41]. Precise patient characterization is impossible using an App due to privacy reasons. Nonetheless, mobile technology is becoming an important tool for better understanding and managing AR. It also provides novel information that was not available with other methods [61, 62, 63, 64, 65, 66, 67]. To our knowledge, there is no other mHealth study that assesses the efficacy of different medications at large scale. 

**Physician’s view**

There are major differences between the physician’s recommendations and the patient’s behaviour in the treatment of pollen-induced AR. Regular use throughout the season, even on days with few symptoms, is generally recommended. In fact, most patients use AR drugs only when needed – if their AR symptoms are not well controlled [41, 68]. An interesting finding is that physicians who suffer from AR behave in the same way as their patients and do not follow the guideline recommendations [69]. 

**Patient’s view**

According to the German Allergy and Asthma Association (Deutscher Allergie- und Asthmabund (DAAB)), a significant part of the problem can be attributed to the inadequate care situation of patients with AR. The worsening in care due to the elimination of reimbursement for antihistamines and INCSs is eminent. For this reason, many patients are not under medical supervision as they have to pay for their own pharmacotherapy and therefore do not see any point in visiting a doctor. As a result, other therapeutic options such as allergen avoidance and early AIT are used too rarely. The DAAB therefore generally calls for the possibility of prescribing over-the-counter anti-allergic drugs at the expense of the statutory health insurances. 

If an allergy is suspected, an early diagnosis should take place, so that patients are aware of their triggers. Furthermore, therapeutic options need to be considered with the aid of allergen avoidance, pharmacotherapy and causal treatment by AIT. The allergy diagnostics should be made by allergologically experienced physicians, possibly with an additional allergologist qualification. An accurate diagnosis of allergy is particularly important in order to decide if patients are eligible for AIT and if a suitable therapy preparation is available for treatment. Molecular component diagnostics for the determination of major allergens is still poorly used in Germany but could further improve the diagnosis and thus the effectiveness of the therapy. Therefore, further studies should be carried out on this diagnostic possibility. In addition, high adherence to the treatment of allergies is necessary for a successful therapy. 

### Next-generation ARIA-GRADE guidelines 

The algorithm proposed a stepwise approach for the selection of AR medications based on GRADE recommendations refined with RWE and chamber studies (Table 1). 

The proposed approach confirms the validity of most GRADE recommendations for AR, allows some conditional evidence to be supported by RWE and provides some new insights. 

In particular:

- The efficacy of combined oral H1-antihistamines and INCSs was not found to be more effective than INCSs alone,
- The efficacy of combined nasal H1-antihistamines and INCSs was found more effective than INCSs alone,
- Intranasal H1-antihistamines are effective within minutes,
- Higher costs of a fixed combination of INCSs and nasal H1-antihistamines are justified if the symptoms cannot be controlled otherwise [3].

The ARIA algorithm for AR was tested with randomized controlled trials (RCTs), observational research RWE and chamber studies. The overall algorithm was found appropriate and no change was needed. 

### Conclusion 

The approach for next-generation ARIA guidelines with the integration of GRADE guidelines, considering RWE and additive studies (pollen chamber exposure studies), could be a model for other chronic diseases as well. The inclusion of ICPs and health apps with integrated, person-centered care represents the ARIA phase 4 change management strategy [18]. 

Special features in the German health care system arise from the OTC availability of most AR drugs and the statutory provision that OTC medicines may only be prescribed in exceptional cases at the expense of the SHI. 

## ARIA care pathways for allergen immunotherapy 

Allergen immunotherapy (AIT) is a proven therapeutic option for the treatment of AR and/or asthma for many standardized products by sublingual (SLIT) or subcutaneous (SCIT) routes [5, 71, 72, 73, 74, 75, 76]. The efficacy of approved AIT products has been demonstrated in double-blind, placebo-controlled, randomized clinical trials (DBPCRCTs) and confirmed in real-life [77]. For AIT, a good patient selection should be made such that indications and contraindications are adequately addressed [1]. [Table Infobox6]

**Figure 8.**
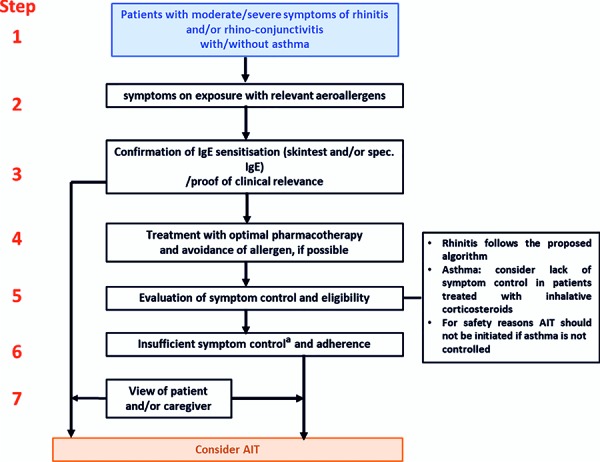
Step-by-step approach to the indication for AIT. Due to the characteristics of a direct access to a specialist in the German health care system, the entire treatment chain from anamnesis to allergen avoidance information, pharmacological therapy, indication and implementation of the AIT, that also can be performed by an allergologically experienced specialist or a physician with additional training in allergology, an early AIT can be enabled. a for exceptions see text. (Reprinted with kind permission from [84])

A major advantage for AR patients in the German health care system is the special feature of having direct access to a specialist (including an allergist). In contrast to many other countries, the entire treatment chain in Germany can be performed by an allergologically competent specialist or a physician with additional allergology training, from anamnesis to allergen avoidance, pharmacological treatment, indication and implementation of AIT (see also Figure 5, 6, 8). Among other things, this enables the early use of AIT, thereby taking advantage of the preventive effects of this form of therapy. 

In many countries, the initial phase of AIT is more expensive than other medical treatments for AR or asthma [42, 78]. In particular, for the German health care system, it has been shown that socioeconomic cost–benefit and cost-effectiveness analyses for longterm effects always favour AIT compared to symptomatic pharmacotherapy for both AR and allergic asthma. AIT is therefore more cost effective in the longer term [79, 80, 81]. Accordingly, an AIT pays off after already 4 – 7 years in terms of cost–benefit aspects in the German health care system [79, 80, 81]. Here, the long-term effect of AIT, which extends beyond the duration of the therapy, is particularly significant. However, such cost-benefit analyses are based on model variables that may include systematic errors [80]. 

Numerous AIT guidelines have been developed [5, 71, 72, 73, 74, 75, 76, 82] and some of the methodologies for evaluating evidence vary considerably. So far, none of these guidelines use ICPs. As requested by an EAACI Task Force [83], ARIA 2019 has created ICPs for both SCIT and SLIT [84], as presented below. 

### Allergens to use 

**Selection of the therapeutic allergen**

The decision to prescribe an AIT should be based on the symptoms of allergen exposure, evidence of sensitization, clinical relevance, and the availability of high-quality therapeutic extracts [71, 85]. 

AIT products must be effective and safe, in accordance with regulatory requirements [86, 87, 88]. Therapeutic allergen extracts cannot be considered generic. In the EU, each AIT product (individual allergens or mixtures) must be tested for its efficacy in a marketing authorization procedure [86, 89] – with the exception for so-called homologous groups, which are allergen sources with a significant clinical cross-reactivity for which defined extrapolations are permissible among each other [86]. In addition, provisions exist in the Directive 2001/83/EC as well as in the German Medicinal Products Act (Arzneimittelgesetz (AMG)), according to which a derogation from the authorization requirement is possible in defined special cases (e.g. for the preparation of a rare therapeutic allergen for a patient, so called a named patient product (NPP)). 

In Germany, as in many other countries, NPPs are used to treat patients individually. The German and European legislation on allergen extracts has created exemptions that make it possible to place these on the market [74, 90]. The details will be discussed in the next section. NPPs that are manufactured using industrial processes should consider both quality aspects and, depending on the frequency of the allergen source, clinical data on a limited scale. A draft version of a position paper on the development of allergen products for which only a few patients are available for clinical trials (concept paper on a guideline for allergen products development in moderate to low-sized study populations) has recently been published by the EMA for public consultation (EMA/712919/2018). Where corresponding RCT studies due to the rare occurrence and insufficiently available patient populations are not possible, RWE studies might under certain circumstances provide clinical data. Due to the importance of these aspects for the availability and selection of therapy extracts, the legal provisions valid for Germany and Europe are presented below. 

**Legal requirements for allergen products in Germany and the European Union (EU)**

Allergens have been subject to European law since 1989 (Directive 89/342/EEC) [91] and, as defined in Directive 2001/83/EC [92], both test and therapeutic allergens are drugs. According to Article 6 of this European Directive, a drug may not be placed on the market in a Member State unless the competent authority of that Member State has granted a marketing authorization [71, 85]. All European Union Member States have at least one national regulatory authority, which cooperates within the network or under the coordination of the European Medicines Agency (EMA) [93]. 

In Germany, the scope of Directive 2001/83/EC has been fully transposed into the German Medicinal Product Act (AMG) [94]. According to § 21 (1) AMG, drugs may only be placed on the market in Germany if they have been granted a marketing authorization by the competent higher federal authority, the Paul-Ehrlich-Institut (PEI) in Langen, which is responsible for allergen products. For marketing authorization, the drugs must be of adequate *quality, efficacy* and *safety* according to the current state of knowledge. The PEI is responsible for the regulation of allergen products based on the applicable national and European legislation and guidelines of the EMA [93]. 

In the European Union there are four different procedures for authorizing a medicinal product [93]:

- National approval procedure: Authorization is sought by the applicant in one Member State (MS). The assessment of the marketing authorization application in the Member State concerned will be carried out by the national competent authority.
- “Mutual Recognition Procedure” (MRP): A national authorization already existing in one Member State (Reference Member State: RMS) may be extended to one or more other Member States at the request of the pharmaceutical company.
- “Decentralized Procedure” (DCP): The applicant seeks simultaneous authorization in several EU countries.
- “Centralized Procedure” (CP): The applicant seeks simultaneous authorization in all EU countries.

Currently, most approvals for allergen products in Germany and Europe are national approval procedures. In Germany, the PEI is the competent federal authority in charge of granting marketing authorization for allergen products. 

**Official batch release**

A characteristic of the German market is the state batch release of therapeutic and test allergens according to § 32 of the German Medicinal Products Act of 24 August 1976 (Federal Law Gazette p. 2445, as amended) [71, 85]. The review and assessment of the PEI is not only based on documentation, but also on the basis of its own experimental tests in the context of state batch release and inspections of license holders and applicants [93]. According to the legislation in Germany, a batch can be released only if the official batch testing has shown that the batch has been manufactured and tested according to state-of-the-art manufacturing and control methods and meets the required level of quality, efficacy and safety. 

With the official batch release testing of allergen products, the Paul-Ehrlich-Institut contributes significantly to ensuring the efficacy and safety of allergen products on the German market. 

**Named patient products and therapy allergen regulation**

According to the European Directive 2001/83/EC, there are various exemptions from the authorization requirement for drugs. Thus, under Article 5 of Directive 2001/83/EC, a Member State may exempt drugs from the provisions of this Directive in specific circumstances, in accordance with applicable legislation (e.g. for individualized drugs). The AMG valid in Germany also contains an exception according to §21 (2). An authorization is not required for drugs that (...) “*are therapeutic allergens manufactured to order for individual patients*” [71, 85, 93]. This exemption is useful and important for the availability of allergen-specific immunotherapies for allergies to rare allergens [93]. 

**Mixing therapy allergen extracts**

There is no evidence that the mixing of different allergens has the same effect as the separate administration of individual allergens. Mixing allergen extracts may result in a dilution effect and an allergen degradation due to the enzymatic activity of certain allergens [95]. For allergen mixtures that do not belong to the same homologous group, the EMA demands a separate justification [86]. A recent report from an NIH sponsored international workshop for AIT on aeroallergens presents study concepts to address this important knowledge gap [96]. 

**Polysensitized patients**

Allergic diseases are complex and diverse. Patients are often simultaneously sensitized to multiple allergens (polysensitization), but not all these sensitizations may be clinically relevant. Therefore, it is important to use only those therapeutic allergens that are directed against the proven symptom-causing sensitization for the AIT and not against a clinically irrelevant sensitization. AIT with single extracts is effective in polysensitized patients [97, 98, 99]. Therefore, it makes sense to use different (mono) allergen extracts separately in polysensitized patients instead of mixing extracts [75]. In Germany, mixing therapeutic allergens is not possible with the Therapy Allergen Ordinance (Therapieallergene-Verordnung (TAV)) for the frequent allergen sources defined herein, since any mixture of these therapy allergens is required to undergo a marketing authorization process. As a result, the number of available mixtures has decreased sharply. When multiple therapy extracts were used in parallel, it was suggested to administer the extracts at different injection sites with a 30-minute interval. However, only few confirming data exist for this procedure. 

**The costs of AIT in the German statutory health insurance (SHI)**

The prescription of therapy extracts for specific immunotherapy in the SHI physician sector, like all forms of therapy, must be based on the specifications of the German Medicinal Products Act. The specifications of the economic efficiency requirements according to § 12 SGB V and the guidelines of the Federal Committee of Physicians and Health Insurance Funds on the prescription of drugs in medical care (AMR) both regulate therapy within the SHI. Recommendations on the economic prescription usually refer to the price list of AIT products [80]. 

The real prices of the products, massively influenced by current legal framework conditions, are often ignored in this field [100]. Therefore, the price list and the real price tend to differ widely, with a significant impact on the actual costs of AIT. 

Since April 2014, all AIT manufacturers are governed by § 130a (1) SGB V to an amended mandatory rebate of 7% on the price list [100]. This compulsory levy is the same for all reimbursable products. But much stronger affects a so-called price moratorium, which has also been enshrined by law until 2022 (§ 130a (3a) SGB V and AM-VSG). This price moratorium, which came into effect in July 2010, froze all prices at the time of 31 July 2009 [100]. All price increases since this date have subsequently been reclaimed by the health insurance companies via the pharmacy computer centres. This amount, known as the “manufacturer’s discount”, must be refunded by the manufacturer to the respective health insurance company [100]. Therefore, the manufacturers are currently obtaining only the prices that were valid for their preparations on July 31, 2009, further reduced by a mandatory discount of 7% [100]. 

In addition, these significant discounts are not the same for all AIT products. Due to different increases in raw material prices and other costs since 2009, there were very different price increases on the part of the manufacturers. Thus, a look at officially available price lists reveals a highly distorted picture which significantly affects the economics of immunotherapy. This means that the treatment is much cheaper than suggested by the price list. Of course, for all price comparisons, there are preparation-specific differences, e.g. fill volume of the vials, injection volumes, injection intervals, up-dosing schemes, making it difficult to compare the prices at the annual or 3-year level [80]. 

Thus, the calculation of daily treatment costs (DTCs) – as usual in other areas of indication – is not useful for AIT preparations. In the “Official ATC Code” of DIMDI, there is also no DTC information on AIT preparations [80]. Therefore, it should be kept in mind that the real costs of AIT treatment are (almost) always lower than the costs calculated on the basis of the price lists. However, these reductions vary for different preparations [80]. 

### The patient’s view 

The patient’s view should always be considered in order to enable a tailor-made approach to shared decision making (SDM). In case studies on state of knowledge, awareness as a therapy option, expectations and satisfaction with the AIT, there were sometimes very different assessments between the physician’s view and the patient’s view [101, 102]. Most studies complain about a lack of information on the patient side. Therefore, every effort should be made to improve communication between the physician and the patient, thus contributing to a better understanding and patient satisfaction [103, 104]. Before initiating an AIT, patients should be informed about the procedure, type and duration of treatment, expected effects, potential risks and possible alternatives. The Physician’s Association of German Allergists (AeDA) has recently given a comprehensive statement on this topic [105]. 

This self-determination for consent to a medical procedure according to § 630e BGB (1) (sentences 1 and 2) determines the cooperation of the patient with the knowledge of the essential circumstances of the treatment. In particular, this includes information on the nature, extent, implementation, expected consequences and risks, the measure and its need, urgency, suitability and chances of success in terms of diagnosis or therapy. This enables shared decision-making in the sense of the SDM and should be applied from a medical-legal perspective using current medical knowledge on treatment options, risks and benefits [105, 106]. 

According to the German Allergy and Asthma Association (Deutscher Allergie- und Asthmabund (DAAB)), the indication for AIT in AR, especially in childhood and adolescence, should be generous in order to reduce the risk of allergic asthma [72, 107]. Here, the RKI and EAACI’s demand for early causal treatment of hay fever is supported, as the risk of a change in level from AR to allergic asthma is apparently at its greatest when children are young and developing AR [22]. 

Adherence to allergen immunotherapy (AIT) is critical to its efficacy. A SCIT requires regular (usually monthly) visits during the maintenance phase, while a SLIT is performed with a daily intake of allergy tablets or drops at home. Noncompliance with an AIT schedule and premature termination of therapy are common problems [108]. There are controversial results on termination rates in AIT – but overall adherence is low [109]. A good organization plan by allergists not only increases safety, but also provides the ability to accurately track and improve patient adherence and compliance [108]. 

### The pharmacist’s view 

Most patients treat their AR without any interaction with their physician [110]. Pharmacists are the most accessible health professionals to the general public and AR is one of the most common diseases managed by pharmacists [111, 112]. Due to the large number of OTC products for AR, pharmacist consultation plays a key role for most pharmaceuticals. 

In Germany, AIT products are available only in pharmacies and the pharmacist is an important partner in the entire treatment concept. He/she is involved in both organizational issues of drug procurement as well as in the adequate storage and transport of AIT preparations. He/she may also have essential advisory functions on fundamental issues, such as the importance of AIT in respiratory allergies. In addition, the pharmacist can inform the patient about the risk-benefit balance, as well as the benefits of an adequate therapy duration. 

### The general practitioner’s view

In many European countries, the diagnosis and treatment of allergic diseases takes place in the family practice [113, 114], but an AIT is rarely prescribed there. In Germany, this situation is at least partly different. A high number of specialists combined with close networking between general practitioners (GPs) and specialists could be even more important in the future for good care with AIT. The continuous, accessible and holistic situation of GP treatment is important and can support the identification of allergy patients, enable early diagnosis, and be used for periodic follow-up of allergy patients to assess disease control, treatment adjustments, and patient-centred SDM [115, 116, 117]. But very few general practitioners receive formal basic training in allergology [118, 119]. AIT risks can be minimized when AIT is performed by experienced physicians with well-trained personnel and only suitable patients are treated in an environment with available emergency care facilities for the treatment of systemic anaphylactic reactions [120, 121, 122, 123]. 

### Practical approach to patient selection in AIT 

According to the German S2k guideline, AIT is to be performed by physicians who have either the additional training in allergology or adequate therapy experience and are able to treat emergency adverse drug reactions (anaphylactic shock, severe asthma attack, etc.) [74]. 

Since January 1, 1996, the instructions for use and the summary of product characteristics of the hyposensitization solutions used in Germany must contain the following warning: “Hyposensitizing vaccines for injection may only be prescribed and used by allergological trained or experienced physicians.” (Paul-Ehrlich-Institut, decision of April 5, 1995) [74]. 

In principle, the patient perspective should always be considered in the sense of shared decision-making (SDM). 

Written information (“Therapy Information Sheet”) on the conduct of the AIT and on the handling of possible side effects is available as an appendix in the German S2k [74] guideline and should be made available to the patient. If AIT is performed or continued by another physician after the indication has been given, then close collaboration is required to ensure the consistent implementation and low-risk performance of the AIT [74]. This is especially true for the occurrence of adverse drug reactions (ADR). 

**Selection of suitable patients by molecular component diagnostics**

The approach of precision medicine for the selection of an AIT regime is gaining more and more attention [2, 124, 125, 126]. The determination of allergen component-specific IgE may bring potential benefits in the indication for AIT, especially in pollen allergies. Patients without sensitization to major pollen allergens are expected to have low or no response to AIT with commercial allergen extracts as these are standardized for their major allergen content [124, 125, 126]. Panallergens such as profiline or polcalcine are mostly clinically not significant but explain false-positive results in skin tests and in in vitro laboratory diagnostics. Sensitization to panallergens is not an indication for AIT [124, 125, 126]. Data from a retrospective study confirm a better success of AIT with pollen allergens in patients with sensitization to major allergens [125]. Other studies show that the additional determination of allergen components led to a change in the decision by the prescribing specialists on AIT in around half of the children with allergic seasonal rhinoconjunctivitis [124, 126]. Further prospective studies as to whether the therapeutic benefit of AIT with pollen allergens including molecular allergy diagnostics can be improved are necessary and still pending. 

A flow chart for the step-by-step approach to the indication of an AIT has been developed (Figure 8; [1, 2]). 

**Rhinitis and rhinoconjunctivitis in adolescents and adults**

Guidelines and various recommendations from experts in AR pharmacotherapy usually suggest the approach summarized in Infobox 1 [3, 4, 5]. All recommended medications are considered safe at the usual dosage, with the exception of first-generation oral H1-antihistamines and depot-corticosteroids that should be avoided [17]. MACVIA has developed a simple algorithm for step-up and step-down management (Figure 6; [32]). 

In children and adolescents with AR, there is evidence from clinical trials that an AIT may reduce the risk of developing asthma [72, 107]. Therefore, the early use of a causal form of therapy in the sense of AIT should be demanded, especially in these patients. 

**Asthma in adolescents and adults**

AIT should not be used in patients with severe asthma. Biologicals in severe asthma and AIT in allergic diseases target two different patient populations. An algorithm for asthma is not yet available. Uncontrolled asthma is still a contraindication for AIT [127]. GINA (Global INitiative for Asthma) has included a SLIT in its treatment recommendations for house dust mite-induced asthma [128]. The summary of product characteristics for the approved SLIT house dust mite tablet [129] shows that (i) the patient should not have had a severe asthma exacerbation within the last 3 months after the onset of AIT, (ii) in patients with asthma and acute respiratory infection, the start of treatment should be postponed until the infection has subsided and (iii) AIT is not indicated for the treatment of acute exacerbations and patients must be informed of the need to consult a physician immediately if their asthma suddenly worsens, (iv) furthermore, AIT against HDM should initially be used as adjunctive therapy for the treatment of anti-asthmatic pharmacotherapy, and the reduction of asthma medication should be carried out step by step under the supervision of a physician according to the management guidelines. So far, only one AIT product has been approved for asthma as main indication in a European procedure. 

**Multimorbidity**

Multimorbidity – the simultaneous presence of more than one disease in a patient – is very common in allergic diseases, and over 85% of patients with asthma also suffer from AR. On the other hand, only 20 – 30% of patients with AR have asthma at the same time. AR multimorbidity increases the severity of asthma [130]. AIT is able to control AR, conjunctivitis, and asthma multimorbidity, which was considered in the marketing authorization for a SLIT HDM tablet [129]. Other atopic disorders, such as atopic dermatitis and/or food allergies due to cross-reactivity of food allergens with inhaled allergens, as well as other known comorbidities (e.g. depression), may increase the disease burden [131, 132, 133]. 

**AIT in children**

AIT in children may have short-term effects like symptom-relieving, anti-inflammatory and drug-saving, as well as positive long-term effects. For specific products, efficacy has been demonstrated in paediatric studies [134] as have long-term beneficial effects [135]. A recent SLIT study [136], an earlier grass pollen SCIT study [137], and a meta-analysis [138] all provided evidence for the products under study that AIT may delay the onset of childhood asthma [137] or prevent the short-term risk of asthma development [138]. The meta-analysis showed a limited reduction in the short-term risk of developing asthma in patients with AR but with unclear benefit over a longer period [138]. In children with AR without asthma, consideration should be given to the possibility of preventing the onset of asthma, although further studies are needed for an unrestricted recommendation [72]. The authors of this article emphasize that only the use of causal and potentially preventive therapy for AR, namely AIT, should be considered at an early stage, especially in children. In children with moderate/severe AR, an AIT should be initiated early if all other conditions are met. Direct specialist access in the German health system, also to an allergist, paediatric allergist or paediatric pulmonologist, facilitates the early use of AIT by utilizing its preventive effects. 

**AIT in elderly patients**

The immunological situation of elderly allergic patients may differ from that of children and younger adults. A limited number of studies have shown that AIT can also be effective in a population of elderly patients [139, 140]. For a universal recommendation, however, more data are required. 

### mHealth in the AIT precision medicine approach 

*The selection* of patients for AIT can be facilitated by electronic diaries accessed via smartphones [19, 20, 41] or other mHealth tools. Such diaries should query the symptoms of AR as well as the drug consumption. For this, they should provide a complete list of medications available in the country for that particular condition. Based on patient-documented data, physicians can assess whether (i) a moderate uncontrolled disease is present, (ii) symptoms are associated with a pollen season or other allergen exposure and (iii) the pharmacological treatment is following the recommendations for uncontrolled symptoms. Physicians can also assess the duration of uncontrolled symptoms and the impact on productivity or academic performance. An electronic clinical decision support system may help in selecting AIT patients in the future [33]. 

**Follow-up of patients with AIT** The same approach can be used to assess efficacy, provided there is a reliable data input, for the progress monitoring and follow-up of AIT patients [80, 83]. 

## Conclusion 

Because of their incidence and chronicity, massive health restrictions for those affected, and the enormous direct, indirect, and intangible costs involved, allergic diseases are a massive social problem for the health systems of many countries, as well as a health economic problem for many national economies. As structured, multidisciplinary care plans, ICPs describe the key aspects of patient care and promote the implementation of guidelines and their application to the healthcare situation. Before many other diseases, ICPs for respiratory diseases (AIRWAYS ICPs) were developed. Digitalized algorithms facilitate the application and improve the effectiveness and safety of the therapy, self-management strategies and resource utilization. 

ICPs can improve the management of both pharmacotherapy and AIT. With the present publication, this international recommendation of ARIA is transferred to the German healthcare situation. 

## Funding 

Article processing charges were provided by the German Society of Allergology (AeDA). 

## Conflict of interest 

C. Bachert reports personal fees from Mylan, Stallergenes and ALK, outside the submitted work. S. Becker reports personal fees from ALK, Allergopharma, HAL Allergy, Bencard Allergy, Sanofi-Genzyme, Thermo Fisher Scientific and B.R.A.I.N AG, grants and personal fees from PARI GmbH, outside the submitted work. T. Bieber reports personal fees from Sanofi, Novartis, AbbVie, Galderma, Pfizer, Lilly, Kymab, outside the submitted work. J. Bousquet reports personal fees from Chiesi, Cipla, Hikma, Menarini, Mundipharma, Mylan, Novartis, Purina, Sanofi-Aventis, Takeda, Teva, Uriach, other from KYomed-INNov, outside the submitted work. R. Brehler reports personal fees form Berufsgenossenschaften, Gerichten, ÄK Nordwürttemberg, ÄK Westfalen-Lippe, ALK, Allergopharma, Allmiral, Apothekerkammer, Astra Zeneca, Bencard, DPC, Gesellschaft zur Förderung der Dermatologischen Forschung und Fortbildung, GSK, HAL, HNO-Gesellschaft, Leti, Novartis, Pohl-Boskamp, Pfleger, Phadia, Update GmbH, Stallergenes, grants from Biotechtools, Genentech, Novartis, Bencard, HAL, AstraZeneca, ALK, outside the submitted work. V. Cardona reports personal fees from ALK, Allergopharma, Allergy Therapeutics, Diater, LETI, Thermofisher and Stallergenes, outside the submitted work. J. Mullol reports personal fees from ALK-Abelló, Sanofi-Genzyme & Regeneron, Menarini Group, MSD, Mitsubishi-Tanabe, Novartis, UCB Pharma, GENENTECH – Roche, grants and personal fees from URIACH Group, MYLAN-MEDA Pharma, outside the submitted work. V. Mahler indicates that the views expressed in this review are the personal views of the author as an expert in the field of allergology and may not be understood or quoted as being made on behalf of or reflecting the position of the respective national competent authorities, the European Medicines Agency, or one of its committees or working parties. H.F. Merk reports personal fees from MEDA, Grünenthal and Coty, outside the submitted work. T. Jakob reports grants, personal fees and non-financial support from Novartis, ALK-Abello, personal fees and non-financial support from Bencard/Allergy Therapeutics, personal fees from Allergopharma, Thermo Fisher Scientific and Celgene, outside the submitted work. M. Jutel reports personal fees from ALK-Abello, Allergopharma, Stallergenes, Anergis, Allergy Therapeutics, Circassia, Leti, Biomay, HAL, during the conduct of the study; personal fees from Astra-Zeneka, GSK, Novartis, Teva, Vectura, UCB, Takeda, Roche, Janssen, Medimmune and Chiesi, outside the submitted work. L. Klimek reports grants and personal fees from ALK Abelló, Novartis, Allergopharma, Bionorica, GSK and Lofarma; personal fees from Boehringer Ingelheim and MEDA, grants from Biomay, HAL, LETI, Roxall, Bencard, outside the submitted work. P. Hellings reports grants and personal fees from Mylan, during the conduct of the study; personal fees from Sanofi, Allergopharma and Stallergenes, outside the submitted work. J. Saloga reports personal fees from ALK-Abelló, Novartis Pharma and Thermo Fisher, outside the submitted work. C. Schmidt-Weber reports grants from DFG, DZL, during the conduct of the study; personal fees and/or grants from Bencard, Allergopharma, Leti Pharma, outside the submitted work. In addition, he has a patent on AIT biomarker. S. Strieth reports grants from Deutsche Forschungsgemeinschaft (DFG), Stiftung Tumorforschung Kopf-Hals, grants Andreas Fahl Medizintechnik-Vertrieb, Atos Medical, Tracoe Medical, Heimomed Heinze, Bromepithetik, Fresenius Kabi and non-financial support from MED-EL AG, personal fees from AurisMedical,Merck Serono, Otonomy, Inc., Nordmark Arzneimittel, Sonofi Genzyme, ALK-Abelló Arzneimittel, outside the submitted work. R. Treudler reports grants and personal fees from Sanofi-Genzyme, personal fees from ALKAbello, Takeda, Novartis, grants fromHautnetz Leipzig, other from Fraunhofer-IZI Leipzig, outside the submitted work. S. Toppila-Salmi reports consultancy for Mylan Laboratories Ltd, ERT Ltd, Roche Products Ltd, outside the submitted work. C. Vogelberg reports grants and/or personal fees from ALK Abello, Allergopharma, AstraZeneca, Boehringer Ingelheim, Bencard Allergy, DBV Technologies, Novartis Pharma and Sanofi Avensis, outside the submitted work. O. Pfaar reports grants and personal fees from ALK-Abelló, Allergopharma, Stallergenes Greer, HAL Allergy Holding B.V./HAL Allergie GmbH, Bencard Allergie GmbH/Allergy Therapeutics, Lofarma, Biomay, Circassia, ASIT Biotech Tools S.A., Laboratorios LETI/LETI Pharma, MEDA Pharma/MYLAN, Anergis S.A., Mobile Chamber Experts (a GA2LEN Partner), Indoor Biotechnologies, GlaxoSmithKline, Astellas Pharma Global, EUFOREA, ROXALL, outside the submitted work. A. Bedbrook, R. Buhl, G.W. Canonica, T.B. Casale, I. Casper, A. Chaker, W. Czarlewski, W. Czech, J. Fischer, K. Nemat, N.G. Papadopoulos, U. Rabe, M. Kopp, D. Larenas-Linnemann, N. Mülleneisen, K. Hörmann, K. Jung, W. Fokkens, T. Fuchs, M. Gerstlauer, E. Hamelmann, J. Ring, W. Schlenter, H. Seyfarth, A. Sperl, T. Spindler, P. Staubach, A. Wallrafen, W. Wehrmann, T. Werfel, H. Wrede and T. Zuberbier declare that they have no competing interests. 

## Open access 

This article is distributed under the terms of the Creative Commons Attribution 4.0 License, which permits unrestricted use, distribution, and reproduction in any medium, provided the original work is properly cited. 

**Abbreviations Abbreviations:** List of abbreviations.

ADR	Adverse Drug Reaction
AEC	Allergen Exposure Chamber
AeDA	Medical Association of German Allergists (Ärzteverband deutscher Allergologen)
AIRWAYS-ICPs	Integrated care pathways for airway diseases
AIT	Allergen Immunotherapy
AMG	German Medicinal Products Act (Arzneimittelgesetz)
AMR	Pharmaceutical Directive (Arzneimittelrichtlinie)
AR	Allergic Rhinitis
ARIA	Allergic Rhinitis and its Impact on Asthma
Aze	Azelastine
BGB	German Civil Code (Bundesgesetzbuch)
CP	Centralized Procedure
DAAB	German Allergy and Asthma Association (Deutscher Allergie- und Asthmabund)
DBPCRCT	Placebo-controlled randomized clinical trial
DCP	Decentralized Procedure
DIMDI	German Institute for Medical Documentation and Information (Deutsches Institut für Medizinische Dokumentation und Information)
DTC	Daily Treatment Cost
EAACI	European Academy for Allergy and Clinical Immunology
EIP	on AHA European Innovation Partnership on Active and Healthy Ageing
EIT	European Institute for Innovation and Technology
EMA	European Medicines Agency
EU	European Union
FP	Fluticasone Propionate
GINA	Global Initiative for Asthma
GP	General Practitioner
GRADE	Grading of Recommendations-Assessment, Development and Evaluation
HDM	House Dust Mite
ICP	Integrated care pathway
INAH	Intranasal Antihistamine
INCS	Intranasal Corticosteroid
J-FC	Joint Federal Committee
LTRA	Leukotriene Receptor Antagonist
MACVIA	MAladies Chroniques pour un Vieillissement Actif (Fighting chronic diseases for active and healthy ageing)
MASK	Mobile Airways Sentinel NetworK
MASK-air	(formerly Allergy Diary)
MPAzeFlu	Nasal fixed combination combining Azelastine and Fluticasone
MRP	Mutual Recognition Procedure
MS	Member State
NPP	Named Patients Product
OAH	Oral Antihistamine
OTC	Over the Counter
PDC	Proportion of Days Covered
PEI	Paul-Ehrlich-Institut
POLLAR	Impact of Air POLLution on Asthma and Rhinitis
RCT	Randomized controlled trial
RKI	Robert-Koch-Institute
RMS	Reference Member State
RWE	Real-world evidence
SCIT	Subcutaneous Immunotherapy
SDM	Shared Decision Making
SGB	Social Security Statute Book (Sozialgesetzbuch)
SHI	Statutory Health Insurance
SLIT	Sublingual Immunotherapy
TAV	Therapy allergen ordinance (Therapieallergeneverordnung)
US	United States
VAS	Visual Analog Scale

**Table 1.** Next-generation ARIA-GRADE guidelines.

	GRADE recommendation	mHealth RWE	Chamber studies
Oral H1-antihistamines are less potent than INCSs BUT many patients prefer oral drugs	[5] No information on the patient’s preference	[41, 59] No information on the patient’s preference	–
Intranasal H1-antihistamines are less effective than INCSs	[5]	[41, 59]	–
Intranasal H1-antihistamines are effective within minutes	[5]	–	[51, 54]
INCSs are potent medications	[4, 5]	[41, 59]	–
The onset of action of INCSs takes a few hours to a few days (except for ciclesonide that is effective quicker)	[5]	–	[53, 70]
The combination of INCSs and oral H1-antihistamines offers no advantage over INCSs	[3, 4]	[41, 59]	–
The fixed combination of INCSs and intranasal H1-antihistamines is more potent than INCSs	YES – in case of moderate to severe symptoms [4]	[41, 59]	–
The fixed combination of INCSs and intranasal H1-antihistamines is effective within minutes	–	–	[16, 53, 55]
Leukotriene antagonists are less potent than INCSs	[4, 5]	–	–

*ARIA* = Allergic Rhinitis and its Impact on Asthma; *GRADE* = Grading of Recommendations -Assessment, Development and Evaluation. (Reprinted with kind permission from [27, 32, 84]).

**Infobox 1.** Legal basis for the exemption from the obligation to prescribe at the expense of the SHI.

*Legal basis*
According to § 34 (1) sentence 1 SGB V, nonprescription medicines are excluded from the supply according to § 31 SGB V. In accordance with § 34 (1) sentence 2 SGB V, the Joint Federal Committee stipulates in the guidelines pursuant to § 92 (1) sentence 2 no. 6 SGB V which nonprescription medicines that are considered to be standard therapies for the treatment of serious illness are to be used for these diseases may exceptionally be prescribed by the contract doctor. In doing so, account must be taken of therapeutic diversity (§ 34 (1) sentence 3 SGB V).
According to § 34 (1) sentence 5 SGB V, exclusion under sentence 1 does not apply to
1. insured children until the age of 12 years,
2. insured adolescents up to the age of 18 with developmental disabilities.
The legal criteria are specified in § 12 (3) and (4) of the current Medicines Directive as follows:
- § 12 (3) A disease is serious if it is life-threatening or if, due to the severity of the health disorder caused by it, it permanently affects the quality of life.
- § 12 (4) A pharmaceutical product is considered to be the standard of care if the therapeutic benefit for the treatment of the serious disease is in line with the generally accepted state of medical knowledge.

**Infobox 2.** Recommendations for pharmacotherapy for allergic rhinitis.

- Oral or intranasal H1-antihistamines are less effective in controlling all rhinitis symptoms than intranasal corticosteroids (INCSs) [5, 6, 7, 8, 9, 10]. However, they are effective in many patients with mild to moderate disease and many prefer oral medication.
- The comparisons between oral and intranasal H1-antihistamines differ in their results; no final conclusions have been drawn.
- In patients with severe rhinitis, INCSs are the first-choice in treatment. Onset of action takes place after a few days.
- The concomitant use of an oral H1-antihistamine and an INCS does not provide better efficacy than INCSs alone [3, 4], although this is a common practice worldwide.
- MPAzeFlu, the fixed combination of intranasal FP and azelastine (Aze) in a nasal spray, is more effective than INCS or H1-antihistamine monotherapy and is indicated for patients in whom INCS monotherapy is considered inadequate [11, 12, 13, 14, 15], with severe AR or for patients who want a quick relief of symptoms [3, 4]. In a pollen exposure chamber study, the speed of onset of the combination was confirmed [16, 17].
- All recommended medications are considered safe in the usual dosage. Oral H1-antihistamines of the first generation are sedating and should be avoided [17], as well as the prolonged use of nasal alpha-sympathomimetics (in vasoconstrictive nasal sprays).
- Depot corticosteroids i.m. are not indicated in allergic rhinitis.

**Infobox 3.** General recommendations of ARIA 2017 [3].

1. In patients with seasonal AR, INCSs are recommended, or possibly a combination of INCSs + OAH. But the potential added benefit has not been proven.
2. In patients with persistent AR, INCSs alone are recommended rather than a combination of INCSs + OAH.
3. In patients with severe seasonal AR, a fixed combination of INCSs+ INAH or INCSs alone is recommended; the choice of therapy also depends on the patient’s preferences. At the beginning of treatment (in the first 2 weeks), a fixed combination of INCSs+ INAH will work faster than INCSs alone.

**Infobox 4.** Key clinical advice of US Practice Parameters [4].

For the initial treatment of nasal symptoms of seasonal allergic rhinitis in patients ≥12 years, clinicians:
- should routinely prescribe monotherapy with an intranasal corticosteroid rather than a combination of an intranasal corticosteroid and an oral antihistamine,
- should recommend an intranasal corticosteroid over a leukotriene receptor antagonist (for ≥ 15 years of age),
- for moderate to severe symptoms, the combination of an intranasal corticosteroid and an intranasal antihistamine may be recommended.

**Infobox 5.** Results of RWE for the treatment of AR.

1. Patients do not follow guideline recommendations and often treat themselves.
2. Adherence to treatment is poor.
3. Patients treat themselves as needed, depending on symptom control, and enhance their therapy if they feel unwell. However, the concomitant use of arbitrary combinations of various medications does not improve symptomcontrol.
4. MPAzeFlu is superior to ICNSs which are superior to oral H1-antihistamines.

**Infobox 6. Infobox6:** Indication for AIT [1, 2].

1. Accurate diagnosis with medical history, skin test and/or specific IgE and optionally component-based in vitro diagnostic (CRD). In certain cases, provocation tests are required. Approved indications are allergic rhinitis/conjunctivitis and/or allergic asthma.
2. Allergic symptoms must be caused predominantly by the respective allergen exposure.
3. Patient selection: Poor symptom reduction despite adequate pharmacotherapy (according to guidelines) during the allergy season and/or change in natural allergy history. mHealth technologies such as the MASK-air allergy app can be of relevant importance for the selection of patients (mHealth-Biomarkers).
4. Verification of the efficacy and safety of the selected product through appropriate studies. (For therapy allergens containing one or more allergen sources listed in the TAV, at least one DBPC trial with an adequate number of patients and state-of-the-art statistical evaluation proofing positive benefit-risk-ratio is required for granting a marketing authorization.)
5. Shared decision-making considering the wishes of the patient (and the caregiver) are an essential part of the indication.
